# Assessment of the responses of soil pore properties to combined soil structure amendments using X-ray computed tomography

**DOI:** 10.1038/s41598-017-18997-1

**Published:** 2018-01-12

**Authors:** Yonghui Yang, Jicheng Wu, Shiwei Zhao, Qingyuan Han, Xiaoying Pan, Fang He, Chun Chen

**Affiliations:** 1 Institute of Plant Nutrition & Resource Environment, Henan Academy of Agricultural Sciences, Zhengzhou, Henan 450002 China; 2 Yuanyang Experimental Station of Crop Water Use, Ministry of Agriculture, Yuanyang, Henan 453514 China; 3 State Key Laboratory of Crop Stress Biology in Arid Areas, College of Life Sciences, Northwest A&F University, Yangling, Shaanxi 712100 China; 4 Institute of Soil and Water Conservation, Northwest A&F University, Yangling, Shaanxi 712100 China; 5 Armed Police Corps Hospital of Henan, Zhengzhou, Henan 450052 China

## Abstract

Soil amendments, such as straw mulch, organic fertilizers and superabsorbent polymer (SAP), are extensively applied to improve soil structure and porosity, and we reported the functional consequences of the individual application of these amendments in our previous study. However, whether combined amendments are more effective than their individual applications for improving soil pore structure is unknown. Here, we conducted X-ray computed tomography (CT) scanning on undisturbed soil columns to investigate the efficiency of two-amendment application, including straw mulch and organic manure, SAP and organic manure, or SAP and straw mulch, for improving soil pore properties and pore distribution. The X-ray CT technique allows us to accurately determine the number, morphology, and location of macropores (>1 mm in diameter) and smaller pores (0.13–1.0 mm). Compared to the control treatment, which showed the lowest increase in soil porosity, all the combined treatments led to an increase in the numbers of both macropores and smaller soil pores, causing a significant improvement in soil structure and porosity. Among these treatments, the application of both straw mulch and organic manure was the most effective for improving soil porosity and soil physical structure.

## Introduction

Soil structure amendments, such as straw mulch, organic manure, and superabsorbent polymer (SAP), have been widely applied to improve soil organic matter, fertility^1^ and soil structure^2^. For example, Antonio *et al*.^3^ performed a three-year experiment and found that straw mulch can improve several soil structure properties, including soil bulk density and porosity, aggregate stability, effective water content and conductivity. Similarly, Liu *et al*.^4^ reported that no tillage in combination with straw mulch can improve the subsoil structure, where an increase in the equivalent diameter (derived from measurements of soil water suction) of aeration pores was observed at a soil depth of 10–20 cm. However, a reduction in the equivalent aperture of invalid pores was also found. Adding organic matter (e.g., compost or manure) to soil can both elevate the organic carbon content^5,6^ and promote the formation of more and larger aggregates^7^, thereby enforcing the soil aggregate stability and water storage capacity^5,8–10^. Additionally, SAP are widely applied to crop fields as a typical soil amendment to enhance aggregate formation^11^ and improve the soil physical characteristics, such as soil structure, pore properties^12^, capillary porosity, total soil porosity^13^ and soil infiltration^14^. Moreover, the application of SAP performed the additional function of inhibiting the soil crust formation and soil surface evaporation.

Computed tomography (CT) scanning is increasingly used as a non-destructive imaging technique for the high-resolution (mm- to µm-scale) characterization and quantification of soil physical properties^15^. CT scanning has been applied to the study of soil pore properties^16,17^ and pore distribution^18–20^. This technique has also been introduced to accurately measure the number, size, and location of macropores (>1 mm in diameter)^21^. CT scanning has been successfully applied in measurements of the distribution, number, shape and connectivity of macropores in longitudinal and transverse cross-sections of both undisturbed and packed soil columns^12,18,22^. The quantification of pore structure at mm to µm scales has been shown to be important in predicting micro-scale fluid flow properties^23^. Furthermore, the approach is used to quantify the pore distribution of reconstructed soils during ecological restoration in opencast coal mines^24^.

Soil pore structure plays a key role in the movement of water in both topsoil and subsoil, which is closely related to soil surface runoff and permeability^25^, and soil pore morphology affects the transmission and preservation of soil moisture. In soils, irregularly shaped pores usually retain moisture better than circular pores, but the latter can transport water more efficiently. In other words, high soil pore circularity is beneficial for the transport and conservation of water in the soil, which can enhance water utilization and ensure that plants uptake sufficient water. Previous studies have highlighted the influences of pore size on soil water movements. For instance, Lv *et al*.^26^ suggested that pore connectivity is better to facilitate the transmission of water from the pores when the soil pore diameter is less than 0.99 mm. Luxmoore *et al*.^27^ indicated that macropores (>1 mm) promoted rapid soil water movement through the movement of air and water within the soil profile, which is consistent with the findings reported by Perret *et al*.^28^ and Fox *et al*.^29^. Thus, understanding the effects of these soil amendments on soil pore structure is important for the development of effective soil and water conservation and management practices. Soil pore structure is evaluated based on morphological characteristics (e.g., the number, radius, size distribution and circularity of the pores), the distribution of pore spaces, and interactions between connectivity and the spatial pore distribution^19,22^. Our previous study indicated that when soil amendments are used alone, such as straw mulch, SAPs and organic manure, they can significantly improve the characteristics of soil pores and pore distribution in soils^30^. However, little information exists on the changes in soil porosity in response to the combined application of soil amendments, such as straw mulch in combination with organic manure (straw mulch + organic manure), SAP + organic manure, or SAP + straw mulch. The quantitative evaluation of such combined treatments is required to understand their effects on the characteristics and distribution of soil pores. In this study, we hypothesized that the combined application of soil amendments would improve soil porosity by altering pore characteristics within the soil profile. The objectives of this study were to (1) evaluate differences in the CT-measured characteristics (number of pores, porosity, and circularity) of >1.0 mm pores, 0.13–1.0 mm pores and ≥0.13 mm pores (total pores) among treatments and (2) determine whether an observed correlation exists between CT-measured pore parameters and conventional cutting ring approach-derived characteristics (e.g., soil bulk density, field capacity, water-stable aggregate content, and porosity).

## Methods

### Study site

Soil columns were excavated from a field site in Tongxu County, Henan, China (34.429°N, 114.450°E, 60 m.a.s.l.). The study area has a flat topography and 682.4 mm of average annual rainfall. The sandy alluvial soil in this region is derived from river alluvium, with a mechanical composition of 67.8% sand (2–0.02 mm), 18.1% silt (0.02–0.002 mm) and 14.1% clay (<0.002 mm). The field was previously uniformly fertilized and used for growing corn (*Zea may* L.), where the hydrolytic nitrogen (N), available phosphorus (P) and available potassium (K) contents were 55.9, 15.9 and 69.4 mg·kg^−1^, respectively, and topsoil organic matter and total N were 11.9 and 0.85 g·kg^−1^, respectively.

### Field experiment

Based on the results of our previous study^30^, SAP (SNF Co. Ltd, France), organic manure (chicken manure composted for one year), and straw mulch (treated approximately 5 months before the experiment started) were selected as soil amendments in this study. Combinations of two soil amendments were applied to the research fields. A randomized complete block design was used with three replications. The field experiment was performed in wheat-maize rotation croplands from October 2009 to June 2013. The treatments included i) the control treatment (CK, use of chemical fertilizer only, not treated with any soil amendments); ii) straw mulch (SM) (4500 kg ha^−1^ corn straw, 10 mm depth) combined with the application of organic manure (OM) (750 kg ha^−1^, containing 1.5% N, 1.2% P, and 0.8% K), designated *SM* + *OM*; iii) the combined application of SAP (60.0 kg ha^−1^) and organic manure (*SAP* + *OM*); and iv) the application of SAP amended with straw mulch (*SAP* + *SM*), in which the tillage depth was 15 cm during the winter growing season. For the soil background, land history, and soil tillage pattern, the control is consistent with the other amendments treatments. Prior to sowing, all fields received a base fertilizer of ordinary superphosphate (90.0 kg ha^−1^ P_2_O_5_) and N (112.5 kg ha^−1^) and were also supplied with additional N fertilizer at the jointing (45 kg ha^−1^) and grain filling stages (67.5 kg ha^−1^). The assigned fertilizer levels were set with reference to local field managements.

In June 2013, after the wheat harvest, undisturbed soil columns (for the determination of soil pores by CT scanning), ring cut samples (for the determination of soil bulk density, soil total porosity, capillary porosity, non-active porosity and soil field capacity) and undisturbed soil samples (for the determination of water-stable aggregate content) were collected sequentially from the topmost soil (0–13 cm).

### Soil sampling

Undisturbed soil columns were obtained using rigid PVC pipes (inner diameter 50 mm, wall thickness 2 mm, and length 130 mm). Three replicates of each soil column were collected from each sampling site and then stored at 4 °C until further analysis. In order to determine the threshold of pore, two soil columns with known pore diameter were firstly developed. Macropores of a known size were obtained by placing two steel rods (2.0 and 2.4 mm in diameter) in separate PVC tubes. The tubes were filled with soil (0.25 mm sieve size, 2.0–3.5% soil moisture), then was used to measure and ensure the pore threshold under the same bulk density of 1.25 g cm^−3^. Next, the steel rods were then carefully pulled out before the beginning of CT scanning^18,31^.

### CT scanning

A medical 256-speed Discovery ST16 PET/CT scanner (GE Healthcare, United States) was operated to scan soil columns, with a peak voltage current of 120 kV, scanning set of 110 mA and 1 s scanning time. The scanning thickness was 1 mm. Scan depths were set at 25 mm up to 120 mm from the top of the soil column. The scanning thickness was 1 mm, and CT images at 5 mm increments were used for the further analysis of soil pore structure properties. Thus, 20 cross-sectional images, which were two-dimensional image, were obtained for each of the 12 soil columns. A total of 240 CT scan images (20 images × 4 treatments × 3 replicates) were captured. The scanning image brightness represented different soil density areas, and the soil macropores could be clearly displayed from the image^22^. The colour image density of the smaller region was black, while the greater regional colour density was lighter.

### Image analysis

The captured CT scanning data were recorded as greyscale images in PNG format with a size of 50 × 50 mm. These images were processed and analyzed using ImageJ software according to Abramoff’s protocol^32^. Then, the images were converted to 8-bit images and segmented using a threshold value of 56, which was selected on the basis of CT scans of two cores with a known size of macropores^33^. First, we calculated the macro-pore size (macropores of a known size from two soil columns with known pore diameter) based on image analysis results using ImageJ by assuming one threshold value for the threshold sample; then, we compared the obtained size to the actual size. If the difference between the sizes was too large, then we selected another threshold value until the difference was reduced to less than 1%^33^. Segmentation was used to convert the greyscale images into black and white images, which can be used to differentiate between the soil matrix (white areas) and soil pores (black areas). These images were analyzed to determine the pore number, area and perimeter length. The pixel resolution was 0.13 × 0.13 mm (the window size of the CT scanner was 65 × 65 mm, and the image pixel was 512 × 512; thus, the minimum equivalent aperture was 65/512 = 0.13 mm). The X-ray beam width or “slice” thickness was 1 mm, producing a volume element (voxel) size of 0.017 mm^3^. Therefore, soil pores with equivalent pore diameters (≥0.13 mm), based on two-dimensional image (cross-sectional CT image) were reliably identified using this image-processing method. The three-dimensional visualization of soil pore networks in the soil columns was reconstructed via saturated volume rendering using Amira software. In this study, we just used a schematic diagram of 3D view of soil pore structures via one direction to roughly evaluate the effects of different treatments on soil pore properties.

Soil pores based on two-dimensional image were divided into two size classes: >1.0 mm pores^21,27^ and soil pores with an equivalent diameter of 0.13–1.0 mm^30^. Thus, the pore numbers were counted as the number of >1.0 mm pores and 0.13–1 mm pores in a CT image. Pore circularity represents the degree of the shape of the macropores, and higher values of circularity indicate that soil pores are more circular^34^. Porosity was calculated as the area of the image covered by >1.0 mm pores (for >1.0 mm porosity) or 0.13–1 mm pores (for 0.13–1.0 mm porosity) and expressed as a percentage. Pores >0.13 mm (for >0.13 mm porosity) were defined as the sum of >1.0 mm pores (for >1.0 mm porosity) and 0.13–1 mm pores (for 0.13–1.0 mm porosity). Pore circularity was calculated using the following equation:

$$C=4\pi A/{L}^{2}$$

where *C* is circularity and ranges between 0 and 1, *A* is the pore area (mm^2^), and *L* is the pore perimeter length (mm).

### Analysis of soil moisture, porosity, and density

The sampling depth of the soil samples was 0–13 cm. Field moisture capacity^35^, soil bulk density^36^, total porosity, non-active porosity, capillary porosity and aeration porosity were measured using the cutting ring method. The water-stable aggregate content was estimated using the wet sieving method^37^. More details about these methods can be found in the Supporting Information.

### Statistical analysis

Each experiment was conducted in triplicate. The data was presented as mean value ± standard deviation (SD) and analysed by one-way analysis of variance (ANOVA) with the least significant difference (LSD) test using SPSS Statistics Version 10.0 at “*P* < 0.05” and “*P* < 0.01” level. The Pearson correlation coefficient was calculated to assess the relationships between the soil pore parameters and soil characteristics.

## Results

### Changes in average soil porosity and pores circularity

**Figure 1:**
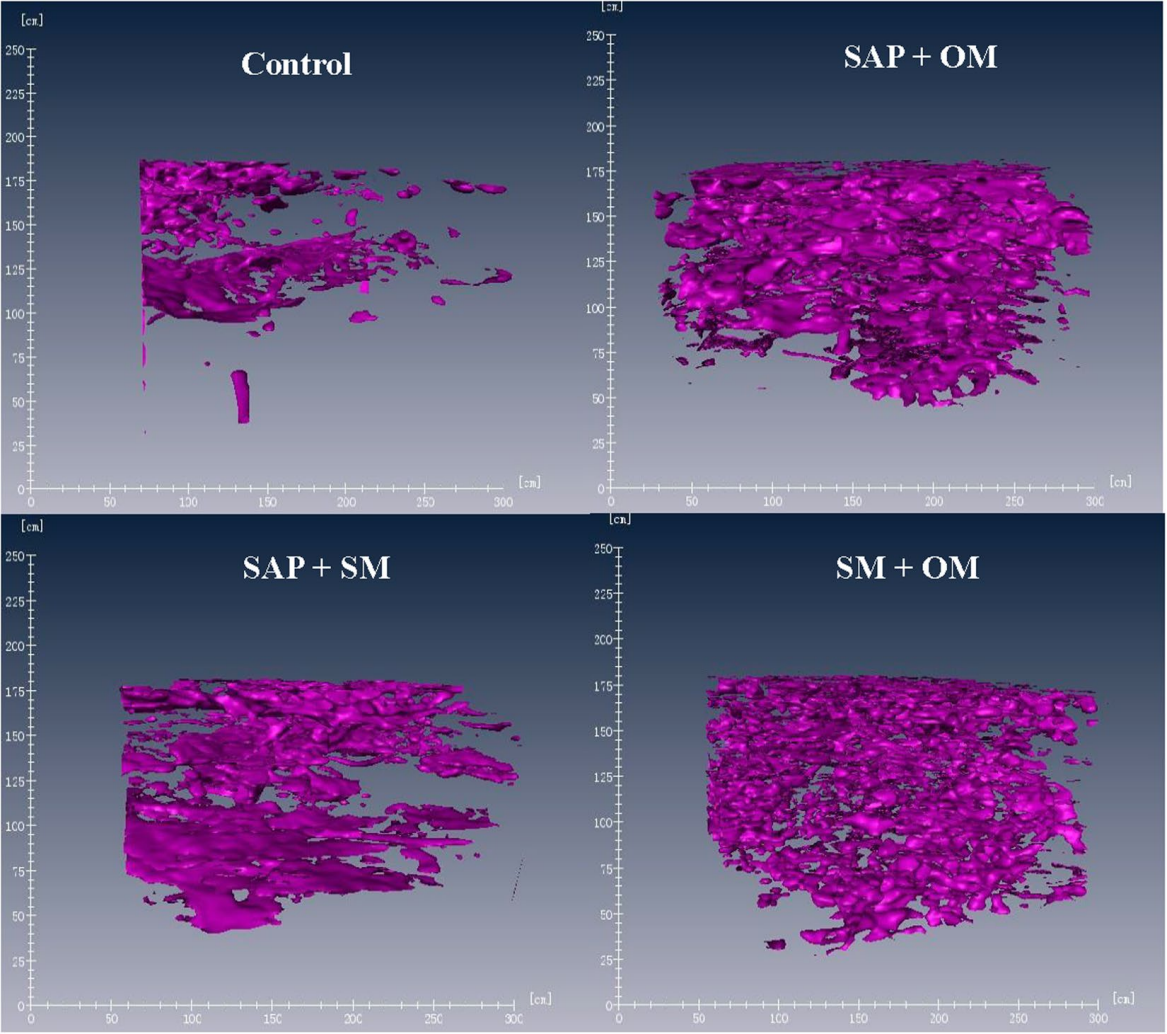
3-D images of soil pore structures detectable on X-ray computed tomography for undisturbed topsoil (0–13 cm). Soil porosity is represented by the purple colour. Control = CK; SAP + OM = superabsorbent polymer + organic manure; SAP + SM = superabsorbent polymer + straw mulch; SM + OM = straw mulch + organic manure. The CT images were captured at 1 mm intervals.

In general, representative 3-D images of soil pore structure from different treatments are presented in Fig. 1. The undisturbed topsoil from the *SM* + *OM* treatment possessed more numerous soil pores and size distribution, clearly showing the general higher soil porosity than other treatments (Fig. 1). The numbers of ≥0.13 mm pores (total pores), >1.0 mm pores and 0.13–1.0 mm pores were significantly increased (*P* < 0.05) in the treated groups relative to the control treatment (CK; Table 1). The *SM* + *OM* treatment caused the greatest number of soil pores with an approximately 3.1-fold higher number of >1.0 mm pores than the control, followed by the number of >1.0 mm pores in the *SAP* + *OM* and *SAP* + *SM* treatments, which were 2.4 and 1.4 times higher than that in the control, respectively. The soil porosity and macroporosity of >0.13 mm pores were the highest in the *SAP* + *OM* treatment, followed by the *SM* + *OM* and *SAP* + *SM* treatments (Table 1), whereas the control treatment produced the lowest level of porosity. The porosity in 0.13–1.0 mm pores was lower than that of the >1.0 mm pores, although the number of 0.13–1.0 mm pores was significantly higher than the number of >1.0 mm pores.

**Table 1:** Treatment effects on the average soil pore numbers, soil porosity and circularity in the topsoil (0–13 cm).

**Treatments**	**Pore numbers**			**Porosity (%)**			**Circularity**		
	**>0.13 mm pore**	**>1.0 mm pore**	**0.13–1.0 mm pore**	**>0.13 mm pore**	**>1.0 mm pore**	**0.13–1.0 mm pore**	**>0.13 mm pore**	**>1.0 mm pore**	**0.13–1.0 mm pore**
Control	17^d^	7^d^	10^d^	4.9^d^	4.7^d^	0.3^b^	0.66^c^	0.45^c^	0.82^b^
SM + OM	55^a^	22^a^	33^a^	11.1^b^	8.4^b^	2.7^a^	0.76^a^	0.61^a^	0.92^a^
SAP + OM	41^b^	17^b^	24^b^	15.3^a^	14.5^a^	0.7^b^	0.73^ab^	0.56^ab^	0.90^a^
SAP + SM	24^c^	10^c^	14^c^	7.1^c^	5.5^c^	0.3^b^	0.71^b^	0.52^b^	0.91^a^

Mean values labelled with the different letters represent significant differences at *P* < 0.05. Control = CK; SAP + OM = super absorbent polymer + organic manure; SAP + SM = superabsorbent polymer + straw mulch; SM + OM = straw mulch + organic manure.

In general, pore morphology affects the movement of air and moisture through the soil. Thus, we compared the pore circularity among the treatments, which describes the morphology of a soil pore relative to a circle with a perfectly circular shape (the value equal to 1.0). For any given pore area, the circularity was reduced as the irregularity of the pore perimeter increased. High pore circularity facilitates water transport in the soil with the increased accessibility. In soil columns among all treatments, the circularity of 0.13–1.0 mm pores was higher than both >0.13 mm soil pores and >1.0 mm pores (Table 1). The circularities of >0.13 mm soil pores and >1.0 mm pores were the highest in the *SM* + *OM* treatment, followed by the *SAP* + *OM* and *SAP* + *SM* (*P* < 0.05) treatments. For the 0.13–1.0 mm pores, the *SM* + *OM* treatment showed the highest circularity, followed by the *SAP* + *SM*, *SAP* + *OM*, and control treatments. Overall, the combined application of the *SM* + *OM* treatment presented the best performance in terms of soil pore circularity.

### Spatial distribution of soil pores at different soil layers

**Figure 2:**
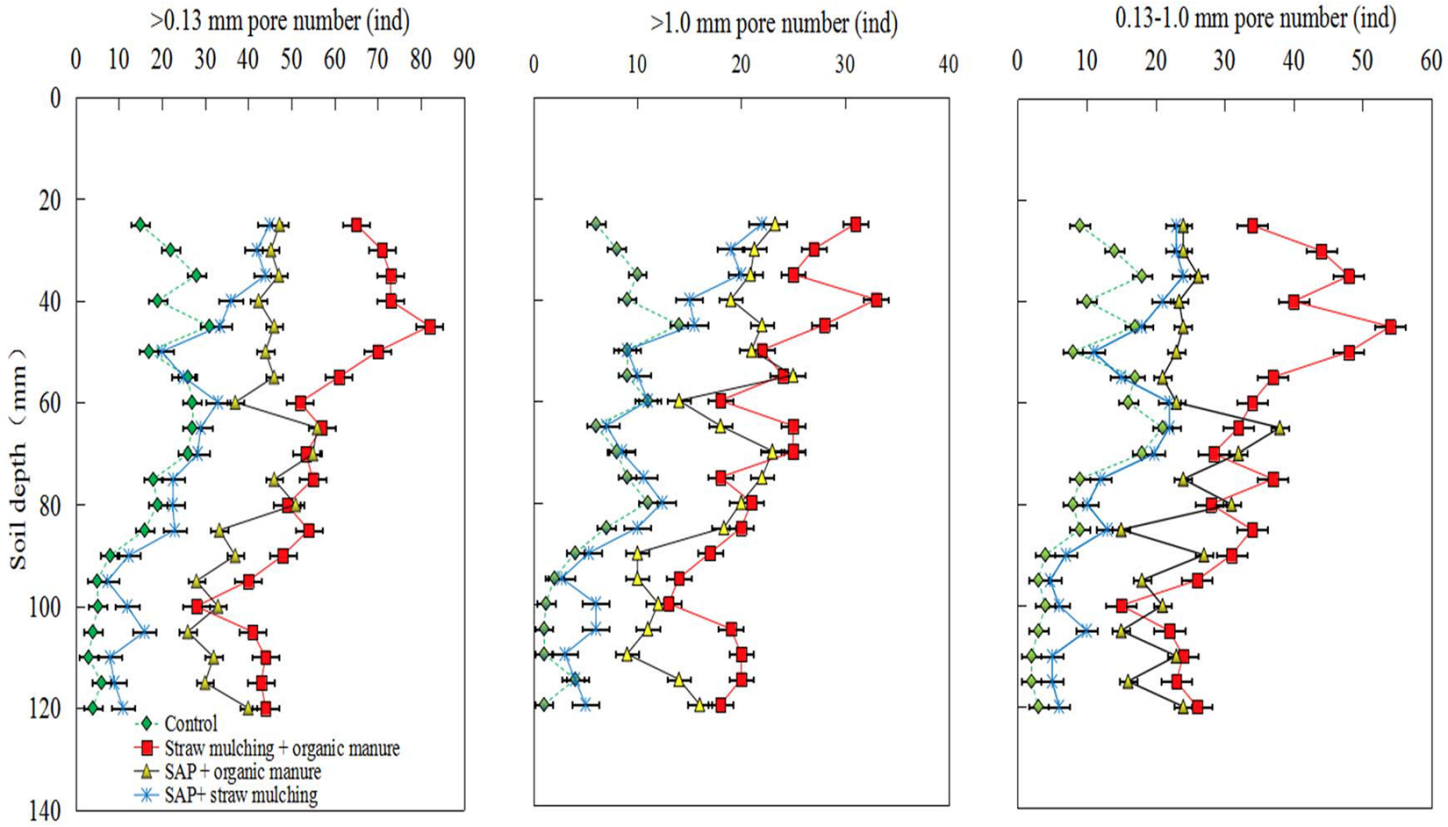
Soil amendment treatment effects on the numbers of >0.13 mm pores, >1.0 mm pores and 0.13–1.0 mm pores in the topsoil (0–13 cm). Error bars indicate the standard deviation of the mean (*n* = 3). CK = Control treatment; SAP + OM = superabsorbent polymer + organic manure; SAP + SM = superabsorbent polymer + straw mulch; SM + OM = straw mulch + organic manure. CT scans are sampled at 5 mm intervals.

Obvious variations in soil pore numbers were observed among treatments across the different soil depths (Fig. 2). In brief, the numbers of >0.13 mm soil pores, >1.0 mm pores, and 0.13–1.0 mm pores generally tended to decrease with increasing soil depth. The control treatment consistently caused the lowest numbers of pores relative to other treatments across all sizes of soils pores. The numbers of >0.13 mm soil pores and >1.0 mm pores, as well as the number of 0.13–1.0 mm pores, at a soil depth of 20–60 mm were significantly increased in the *SM* + *OM* treatment. When the depth of the soil layer was greater than 60 mm, the *SM* + *OM* and *SAP* + *OM* treatments showed similar ≥0.13 mm pore numbers, with a greater number of pores in all size classes than either the *SAP* + *SM* or control treatments. All three combined treatments led to increases in the numbers of both >1.0 mm pores and 0.13–1.0 mm pores.

### Distributions of different sizes of soil pores in different soil layers

**Figure 3:**
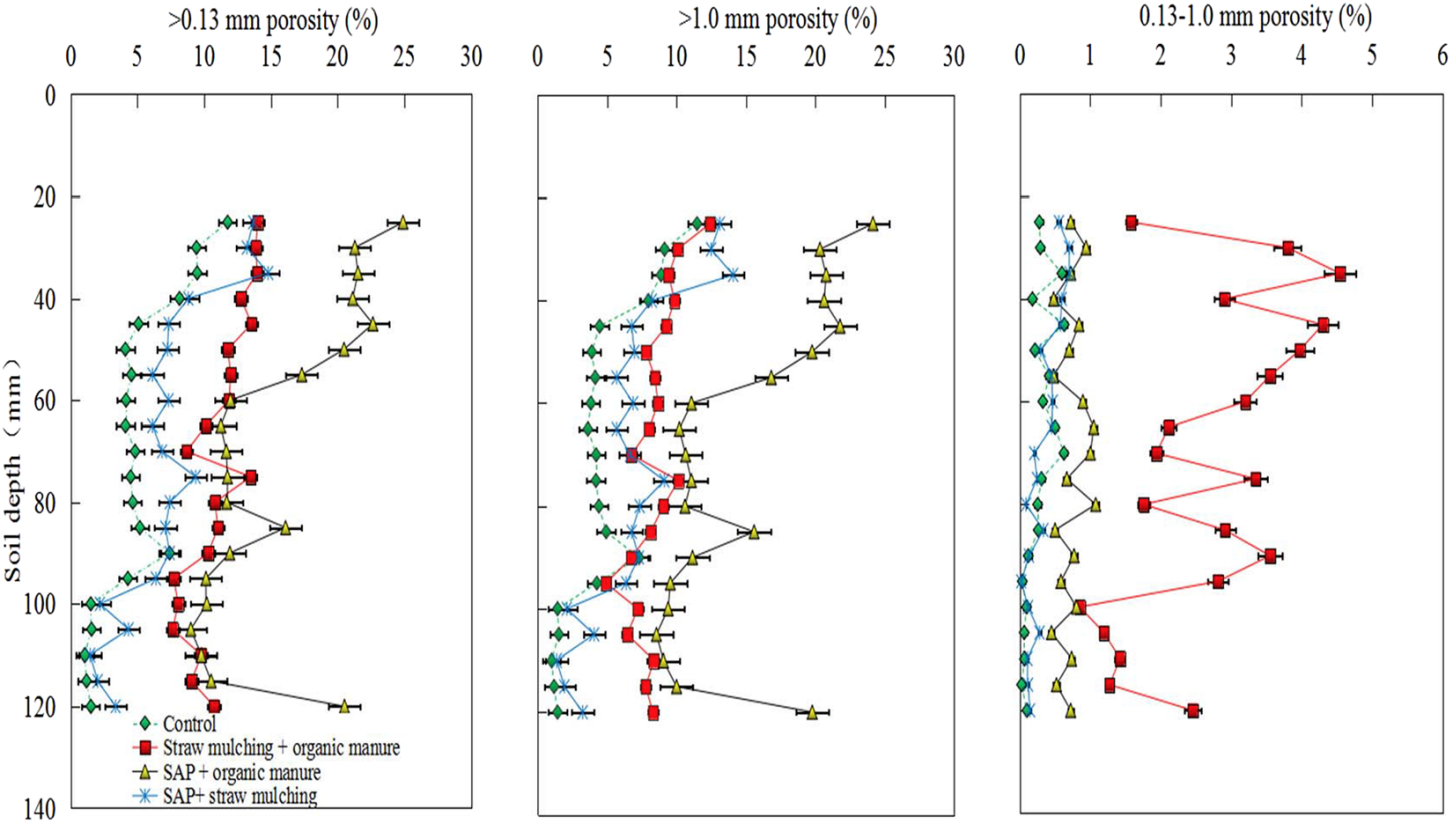
Soil amendment treatment effects on the >0.13 mm soil porosity, >1.0 mm porosity and 0.13–1.0 mm porosity in the topsoil (0–13 cm). Error bars indicate the standard deviation of the mean (*n* = 3). CT scans are sampled at 5 mm intervals.

The soil porosities of ≥0.13 mm soil pores and >1.0 mm pores declined with increasing soil depth in all the treatments, including the control (Fig. 3), whereas, except for *SM* + *OM*, no obvious improvements of the 0.13–1.0 mm porosity were observed among treatments throughout the soil columns. The highest levels of soil porosity for ≥0.13 mm pores were observed in the *SAP* + *OM* treatment at soil depths of 20–75 mm and 80–120 mm. However, for the *SM* + *OM* treatment, the greatest porosity occurred at the 75–80 mm soil depth. Similarly, the varying degree of macroporosity among treatments showed the same pattern of results as the soil porosity of ≥0.13 mm pores, and the highest macroporosity was found in the *SAP* + *OM* treatment throughout the soil columns. Although only a weak difference in soil porosity of 0.13–1.0 mm pores was seen in the *SAP* + *OM* or *SAP* + *SM* treatments relative to the control, a noticeable improvement was found in the *SM* + *OM* treatment. Taken together, these results indicated that the application of the *SM* + *OM* treatment had the greatest benefit for the porosity of 0.13–1.0 mm soil pores, despite the *SAP* + *OM* treatment providing the best overall improvement of soil porosity.

### Distributions of soil pores circularity in different soil layers

**Figure 4:**
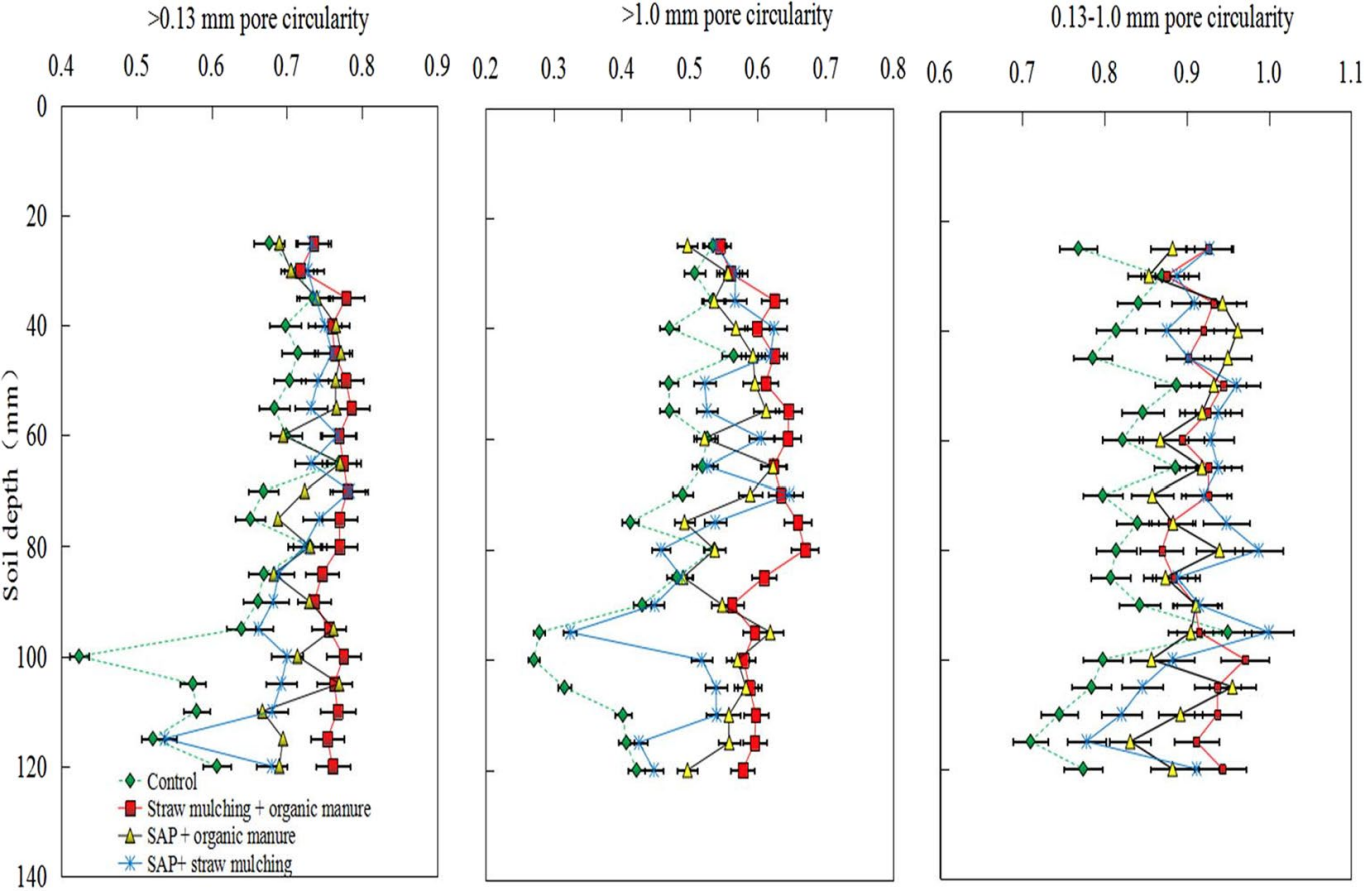
Soil amendment treatment effects on >0.13 mm soil pores circularity, >1.0 mm pores circularity and 0.13–1.0 mm pores circularity in the topsoil (0–13 cm). Error bars indicate the standard deviation of the mean (*n* = 3). CT scans are sampled at 5 mm intervals.

The highest degree of soil pore circularity was observed in the 0.13–1.0 mm pore size class, while the lowest degree was found in >1.0 mm pores (Fig. 4). For all size classes, the *SM* + *OM* treatment led to the highest pore circularity as a whole compared to the control treatment, which showed the lowest pore circularity. When soil pores were obtained from depths of 0–80 mm, pore circularity was elevated with increasing soil depths among all the treatments but decreased with increasing depth at soil depths below 80 mm. Taken together, the depth dependence of the pore circularity was much lower in the *SM* + *OM* treatment than in the *SAP* + *OM* and *SAP* + *SM* treatments. The best pore morphology (i.e., highest pore circularity) was thus provided by the *SM* + *OM* treatment. Consequently, the increases in pore circularity will benefit the soil infiltration capacity as well as the transport and exchange of water and gas.

### Changes in soil bulk density, field capacity and water-stable aggregate content, and porosity

Since the highest level of soil bulk density occurred in the control and the bulk density of a soil was inversely related to the porosity, the lowest porosity caused was anticipated to be present in the control (Tables 1 and 2). The *SM* + *OM* treatment showed the lowest soil bulk density, while the *SAP* + *SM* and *SAP* + *OM* treatments contributed to intermediate soil bulk densities. Compared to the control treatment, all the combined treatments showed significantly higher levels of field capacities (*P* < 0.05, Table 2), while the increasing rates relative to the control were *SAP* + *OM* (25.9%) < *SAP* + *SM* (35.8%) < *SM* + *OM* (53.8%). Given the data from the standard cutting ring approach, the total porosity, capillary porosity and non-active porosity were the highest in the *SM* + *OM* treatment, followed by the *SAP* + *SM* and *SAP* + *OM* treatments, but notably low levels of aeration porosity were found in all the combined treatments compared to the control (Table 2).

**Table 2:** Soil bulk density, field capacity, water-stable aggregate (>0.25 mm) content and porosity measured via the cutting ring method in the topsoil (0–13 cm) for each treatment.

**Treatment**	**Bulk density** **(g cm** ^**−3**^ **)**	**Field capacity** **(g g** ^**−1**^ **)**	**Aggregate (>0.25 mm) content** **(%)**	**Total porosity** **(%)**	**Non-active porosity** **(<0.002 mm) (%)**	**Capillary porosity** **(0.002–0.02 mm) (%)**	**Aeration porosity** **(>0.02 mm) (%)**
Control	1.47^a^	20.1^d^	48.6^c^	44.5^c^	5.3^c^	22.1^d^	17.1^a^
SM + OM	1.34^c^	30.9^a^	66.4^a^	49.3^a^	10.6^a^	27.6^a^	11.1^c^
SAP + OM	1.43^b^	25.3^c^	62.1^b^	45.9^c^	9.7^b^	23.4^c^	12.9^b^
SAP + SM	1.40^b^	27.3^b^	64.9^a^	47.1^b^	10.2^b^	24.8^b^	12.1^b^

Mean values labelled with different letters represent significant differences at *P* < 0.05.

### Correlation analysis between soil pores parameters and soil characteristics

Field capacity as well as water-stable aggregate contents showed a significant positive correlation with circularity (*P* < 0.01), which had a stronger correlation than pore number (*P* < 0.05) among all sizes of soil pores (Table 3). For the different pore sizes, a significant correlation was found only between field capacity and 0.13–1.0 mm porosity (*P* < 0.05). Bulk density showed strong negative correlations with all three pore parameters (*P* < 0.05 or *P* < 0.01), apart from soil porosity in 0.13 mm pores and >1.0 mm pores, which had a weakly negative correlation. For circularity, >0.13 mm pores (*P* < 0.01) exhibited a highly significant negative correlation with bulk density compared to >1.0 mm pores or 0.13–1.0 mm pores (*P* < 0.05). For porosity, there was also only a significant negative correlation between the porosity in 0.13–1.0 mm pores and bulk density (*P* < 0.05). Given the data from the cutting ring approach, the level of total porosity showed a strong positive correlation with pore numbers in >0.13 mm pores and 0.13–1.0 mm pores, porosity in 0.13–1.0 mm pores, and circularity in all sizes of soil pores (*P* < 0.05), the data of which were obtained from the CT scanning technique. A similar correlation pattern was found between the capillary porosity and these soil pore parameters. Non-active porosity levels had a significantly positive correlation with the circularity in >0.13 mm pores and >1.0 mm pores (*P* < 0.05) and a notably positive correlation in the 0.13–1.0 mm pore size (*P* < 0.01). In contrast, aeration porosity had a negative correlation with these pore parameters derived from CT scanning, in which the circularity of 0.13–1.0 mm pores had the most significant effect (*P* < 0.01), followed by >0.13 mm pores and >1.0 mm pores (*P* < 0.05).

**Table 3:** Correlation coefficients between soil pore parameters determined by CT scanning and soil characteristics.

**Factors**	**Pore number**			**Porosity**			**Circularity**		
	**>0.13 mm pore**	**>1.0 mm pore**	**0.13–1.0 mm pore**	**>0.13 mm pore**	**>1.0 mm pore**	**0.13–1.0 mm pore**	**>0.13 mm pore**	**>1.0 mm pore**	**0.13–1.0 mm pore**
Field capacity	0.7958^*^	0.7941^*^	0.8036^*^	0.442	0.0794	0.7680^*^	0.9513^**^	0.9198^**^	0.9213^**^
Aggregate content	0.7018^*^	0.7283^*^	0.6984^*^	0.5617	0.1352	0.5494	0.9191^**^	0.8795^*^	0.9962^**^
Bulk density	−0.7861^*^	−0.7654^*^	−0.8036^*^	−0.3065	−0.0004	−0.8505^*^	−0.9027^**^	−0.8748^*^	−0.8134^*^
Total porosity	0.6180^*^	0.5858	0.6457^*^	0.0939	0.0000	0.7233^*^	0.8184^*^	0.7653^*^	0.6617^*^
Capillary porosity	0.6126^*^	0.5718	0.6439^*^	0.0726	0.0002	0.7630^*^	0.7775^*^	0.5945	0.5945
Aeration porosity	−0.5604	−0.5935	−0.5582	−0.3213	−0.0247	−0.3775	−0.8924^*^	−0.8272^*^	−0.9822^**^
Non-active porosity	0.4982	0.5427	0.4897	0.3653	0.0328	0.2792	0.8426^*^	0.7767^*^	0.9992^**^

Values labelled with “*” and “**” denote a significant difference at *P* < 0.05 and *P* < 0.05, respectively.

## Discussion

In general, a healthy soil consists of a combination of a well-aggregated soil matrix and a well-developed soil pores system, which will provide sufficient aeration for plants and other organisms, a high water retention capacity, and other important soil functions^38^. Our previous work demonstrated that the individual application of SAP, organic manure or straw mulch can remarkably increase the pore number, porosity and pore circularity of various soil layers and thus enhance the crop yields^12,30^. In the present study, we observed that the combined application of two soil amendments was more effective in promoting soil pore structure than these amendments used alone. All three combined amendments were found to significantly enhance the soil field moisture capacity, water-stable aggregate content, total porosity, capillary porosity and non-active porosity, but they diminished the soil bulk density. Furthermore, the combined amendments produced a marked increase in the pore numbers across three sizes of soil pores, and the porosity levels were elevated at the soil depths of 0–60 cm, in which the porosity was highest in 0.13–1.0 mm pores. Moreover, combined amendments had a more pronounced effect on the soil pore circularity.

Soil pore structure exerts a profound influence on water movement between topsoil and subsoil, which is closely related to surface runoff and soil permeability^25^. Of the soil amendments tested, the application of straw mulch in combination with organic manure (*SM* + *OM*) exerted the greatest effect on the improvement of soil pore structure, providing the greatest capacity to potentiate plant growth. As noted by Acharya *et al*.^39^, straw mulch is widely applied to increase soil humus content and promote soil particle aggregation, leading to a more effective improvement of the soil pore structure. Other recent studies have suggested that both straw mulch and organic matter can increase soil organic carbon (SOC), which greatly affects the soil pore distribution^6,38^. This finding is also consistent with a study by Schlüter *et al*.^40^ that reported that higher levels of SOC had a distinctive effect on the quality of the soil structure. Moreover, organic manure was suggested to improve the soil cluster structure through the elevation of soil organic carbon, humus content and aggregates^41,42^. Muhmmad *et al*.^43^ observed a more aggregated structure in the soils where both animal manure and inorganic fertilizer (NPK) had been applied compared to soils fertilized with animal manure alone. In comparison to inorganic fertilization, organic fertilization showed that the soil physical quality can be improved at different scales^44^, as reflected by the increases in pore numbers and porosity^30^. Furthermore, the long-term application of organic manure can improve the macro-pore to secondary pore ratio, which reduces the proportions of smaller pores.

In addition to the size and quantity of soil pores, porosity is another important factor affecting the transmission and preservation of soil moisture. Zhao *et al*.^45^ investigated the effects of mulching pattern on soil physical properties and crop yield in a wheat-maize cropping system and found that straw mulch can enhance soil porosity and reduce the ratio of capillary to non-capillary porosity. Our data are also in agreement with previous reports showing that *SM* + *OM* treatment caused a higher increase in pore numbers and porosity levels at a soil depth of 20–60 mm than in other treatments. The soil pore numbers and porosity in combined amendment treatments were still higher than those of the control, although the beneficial effects on soil structure were attenuated with increased soil depths.

In addition, soil pore shape has been well documented to play an important role in the transmission and preservation of soil moisture. When the pore shape is more irregular, the water transport process in soil pores is slower than circular pores. Hence, maintaining higher soil pore circularity is critical for soil water transportation and conservation. Our study showed that the improvement of the pore morphology in different soil layers as well as the soil pore circularity in the *SM* + *OM* and *SAP* + *OM* treatments was better than in the other treatments. Deurer *et al*.^46^ analyzed the effects of the long-term application of organic carbon on apple orchards using X-ray microtomography and reported that organic carbon affected the soil structure, which had significantly greater macroporosity than in conventionally managed soils. Moreover, SAP was applied to trigger large soil aggregate formation^11^. Our previous studies supported the role of SAP application in improving the soil structure and pore properties via the shrinking/swelling process, agglutination of the soil particles, and reduced infiltration^12,14^. Furthermore, our results suggested that the increase in water-stable aggregation may improve the soil physical properties and make soils more suitable for crop growth, augmenting the soil total porosity, capillary porosity, non-active porosity and soil field capacity and diminishing the soil bulk density and aeration porosity. The cutting ring method-derived properties (including water-stable aggregate content, field capacity, total porosity, capillary porosity and non-active porosity) were distinctly positively correlated with soil pore number, pore circularity and porosity of 0.13–1.0 mm soil pores measured via the X-ray CT technique, which highlights the importance of soil pore structure for soil moisture content. The soil bulk density and aeration porosity were significantly negatively correlated with the cutting ring method-measured parameters, indicating that bulk density or aeration porosity was linked to both the number and morphology of soil pores. Taken together, these findings show that the macroscopic physical characteristics can be reliably represented for the micro-scale physics of soil pore structure, which indicates the potential for translating micro-scale soil processes to macro-scale soil properties. In addition, the quantification of pore structure is more effective in predicting fluid flow properties^23^. Luo *et al*.^47^ found that X-ray CT-derived macroporosity and path numbers are important to effectively predict saturated hydraulic conductivity. Thus, quantitative relationships between X-ray CT-derived soil pore geometry characteristics and flow and transport parameters require further investigation.

## Conclusions

In this study, we combined the X-ray CT scanning of undisturbed soil columns with standard cutting ring measurements to investigate how the combination of two amendments affects soil pore properties and pores distribution. The results clearly reveal that the application of two soil amendments was more effective than a single amendment in the improvement of soil pore parameters, represented as pore number, porosity and circularity. Our results obtained from cutting ring approaches suggest that the application of a combination of two soil amendments is a good strategy to synergize the effects of organic matter to improve the field capacity, total porosity, capillary porosity and non-active porosity and reduce the bulk density and aeration porosity. Correlation analysis results indicate the potential for translating micro-scale soil processes to macro-scale soil properties. The findings show the immense potential of linking X-ray CT-derived soil pore parameters with classical soil physical measurements for quantifying soil structure and functions. Across all treatments in this study, the combined application of straw mulch and organic manure led to the best overall improvement in soil structure and porosity. Further efforts should focus on the development of high-resolution X-ray CT or micro-CT for the investigation of soil physical structure and should consider improvements in the sample size, image contrast and resolution.

## Electronic supplementary material

Supporting Information
