# Supplementary material for: Assessment of the responses of soil pore properties to combined soil structure amendments using X-ray computed tomography
Source: Sci Rep. 2018 Jan 12;8:695. doi: 10.1038/s41598-017-18997-1 (PMC5766547; doi:10.1038/s41598-017-18997-1)
Supplement: Supplementary file 1 — Supporting Information [file 41598_2017_18997_MOESM1_ESM.pdf]

Supporting Information

Assessment of soil pore properties responses to combined soil structure amendments using X-ray computed tomography

Yonghui Yang[1,2*], Jicheng Wu[1,2], Shiwei Zhao[4], Qingyuan Han[5], Xiaoying Pan[1,2], Fang He[1,2], Chun Chen[3*]

[1] Institute of Plant Nutrition & Resource Environment, Henan Academy of Agricultural Sciences, Zhengzhou, Henan 450002, China

[2] Yuanyang Experimental Station of Crop Water Use, Ministry of Agriculture, Yuanyang, Henan 453514, China

[3] State Key Laboratory of Crop Stress Biology in Arid Areas, College of Life Sciences, Northwest A&F University, Yangling, Shaanxi 712100, China

[4] Institute of Soil and Water Conservation, Northwest A & F University, Yangling, Shaanxi 712100, China

[5] Armed Police Corps Hospital of Henan, Zhengzhou, Henan 450052, China

*Corresponding author

Dr. Chun Chen

State Key Laboratory of Crop Stress Biology in Arid Areas

College of Life Sciences, Northwest A&F University

Email: chennchun@yahoo.com and email: yangyongh@mails.gucas.ac.cn

Content

Supplementary Methods and Analyses: The detailed methods to analyse field moisture capacity, non-active porosity, capillary porosity, aeration porosity, and aggregate stability S1

S1 The detailed analyze methods of field moisture capacity, non-active porosity, capillary porosity, aeration porosity, and aggregate stability

Pore are divided into three types according to the soil water suction: I. non-active porosity, also known as invalid porosity, irreducible water pores and micro pores in the soil, which is the most fine pore, the equivalent diameter of less than 0.002mm, and root hair and microorganisms cannot enter the pore; II. capillary pore, equivalent pore diameter is 0.02 ~ 0.002mm, because the capillary action of this pore is very strong, so called capillary pores; III. aeration pore, equivalent pore diameter >0.02mm, under the action of gravity, water rapidly discharges soil or groundwater to recharge groundwater, called aeration pore, the number of which determines soil permeability[1,2].

Soil bulk density: A steel cutting ring was used to obtain a certain volume of soil under the natural condition, which was brought back to the laboratory for the determination of soil dry weight in the cutting ring. The ratio of the soil dry weight to the cutting ring volume was the soil bulk density[3].

Soil bulk density ($g \cdot cm^{-3}$) = dry soil weight in cutting ring/cutting ring volume (1)

Field capacity: Using the centrifuge method in the determination of the soil water characteristic curve, we determined the water content from the soil water characteristic curve, which was the field capacity when soil water suction is 1/3 ba. When the soil water suction was 15 ba, the water content from the soil water characteristic curve was the wiltingpoint[4].

Loosely bound water: The water layer maintained by the adsorption of soil particles is called loosely bound water, and its pores are loosely bound water pores.

Tightly bound water: The water in the dry soil that holds from the air is called tightly bound water, and its pores are tightly bound water pores, also known as hygroscopic water,

Maximum hygroscopicity: A 100 g dry soil sample was placed in moist air to absorb the moisture in the air. When soil was saturated (the soil weight was no longer increasing), the weight of the soil exceeding 100 g was hygroscopic moisture or the coefficient of moisture absorption.

Loosely bound water porosity (%) = (the wilting point-maximum hygroscopicity) × soil bulk density /1.25 (2)

Tightly bound water porosity (%) = maximum hygroscopicity × soil bulk density/1.5 (3)

Total soil porosity (%) = (1 – soil bulk density / soil density) × 100% (4)

Capillary porosity (%) = (field capacity – wilting moisture content) × bulk density × 100% (5)

Non-active porosity (%) = loosely bound water porosity + tightly bound water porosity (6)

Aeration porosity (%) = total porosity – capillary porosity – non-active porosity (7)

Note: The soil density was often 2.65 $g \cdot cm^{-3}$, the maximum hygroscopicity was 3 $g \cdot g^{-1}$, the loosely bound average density was 1.25 $g \cdot cm^{-3}$, and the tightly bound average density was 1.50 $g \cdot cm^{-3}$.

The water-stable aggregate content: An air-dried 100 g soil sample from a 0 - 13 cm depth of undisturbed soil was placed in the aperture of a 0.25 mm automatic oscillating sieve, which was soaked with distilled water for 5 min at room temperature (20 - 25 °C) and shaken for 5 min under the upper and lower amplitude of 3 cm for 30 times per minute. After screening, the aggregate was rinsed from the sieve into the beaker to determine the >0.25 mm water-stable aggregates. The aggregate was poured into the beaker, dried in an oven at 60 °C, and then was weighed.

Reference

[1] Thomasson, A. J. Towards an objective classification of soil structure[. *Journal of Soil Science* **29**, 38-46 (1978).

[2] Skopp, J. Comment on "Micro-, meso-, and macroporosity of soil". *Soil Science Society of America Journal* **45**, 1246-1246 (1981).
[3] Institute of Soil Science, Chinese Academy of Sciences. Determination Methods for Soil Physical Properties. Beijing: Science Press, 1978.
[4] Shi, Z., Zhao, S. The account way of soil water characteristic curve in Loess Plateau. *Acta Agricultural Boreali-Occidentalis Sinica* **8**, 44-47 (1999).
